# Unifying Hypothesis of Dopamine Neuron Loss in Neurodegenerative Diseases: Focusing on Alzheimer's Disease

**DOI:** 10.3389/fnmol.2019.00123

**Published:** 2019-05-17

**Authors:** Paraskevi Krashia, Annalisa Nobili, Marcello D'Amelio

**Affiliations:** ^1^Laboratory of Molecular Neurosciences, Department of Experimental Neurosciences, IRCCS Santa Lucia Foundation, Rome, Italy; ^2^Unit of Molecular Neurosciences, Department of Medicine, University Campus-Biomedico, Rome, Italy

**Keywords:** Alzheimer, dopamine neuron, Parkinson, VTA = ventral tegmental area, degeneration, neuropsychiatric symptoms, midbrain, Tg2576

The term “dementia” describes a syndrome caused by several illnesses in which there is progressive decline in multiple areas of brain function. The main form of dementia is Alzheimer's disease (AD), currently affecting 47 million people, 0.5% of the global population, according to the 2016 World Alzheimer Report (Banerjee, 2012; ADI, 2016). AD is an irreversible and progressive brain disorder characterized by the presence of extracellular amyloid β (Aβ) plaques and intracellular neurofibrillary (tau) tangles, brain atrophy, inflammation and progressive loss of memory and cognition (D'Amelio and Rossini, 2012; Ismail et al., 2016). Comorbidities like epileptic seizures (Noebels, 2011; Pandis and Scarmeas, 2012), neuropsychiatric symptoms, mainly apathy, depression, aggression, and circadian rhythm disturbances (Lyketsos et al., 2011; Masters et al., 2015; Alves et al., 2017; Musiek et al., 2018), impose an additional burden on patients and their families.

There are two forms of AD, early-onset and late-onset (Guerreiro et al., 2012). Early-onset AD represents < 5% of cases and occurs in people aged 30–60. Most patients suffer from the late-onset form, occurring in people over-60. Early-onset AD is strongly linked to autosomal dominant mutations in amyloid precursor protein (*APP*) and presenilin genes [*PSEN1, PSEN2*; (Blennow et al., 2006)] while the most common genetic variant for late-onset AD is apolipoprotein E (*APOE*), a three-allele polymorphism (ε2, ε3, and ε4) where ε4 is the high-risk allele (Corder et al., 1993). However, other than old age, the aetiological factors for late-onset AD remain to be determined, but increasing evidence points to the potential risk roles of vascular diseases and lifestyle factors such as smoking, high blood pressure, high-fat diet, obesity, diabetes, hypercholesterolemia, and cerebrovascular lesions. Conversely, active social engagement, physical exercise, balanced diet, and mentally-stimulating activity have been suggested as beneficial for delaying the clinical manifestation of AD (Kivipelto et al., 2001; Qiu et al., 2009; Mayeux and Stern, 2012; Knight et al., 2014; Winblad et al., 2016).

The broadly-accepted hypothesis of AD pathogenesis states that the generation and accumulation of Aβ peptides represent the cause of the progressive and massive neuronal loss that primarily affects the hippocampus and cortex. Hyperphosphorylated-tau deposition, contributing to neuronal dysfunction and cognitive symptoms, is generally regarded as a downstream event in the amyloid cascade (Hardy and Selkoe, 2002; Scheff et al., 2006; D'Amelio and Rossini, 2012). However, the amyloid cascade hypothesis seems most applicable in cases of early-onset AD, representing a primary cerebral amyloidosis. Whether individuals with late-onset AD also carry genetic variations that promote a primary amyloidosis remains to be shown. Nonetheless, attempts to clear Aβ from the brain led to failure of several long and promising clinical trials (Doody et al., 2013; Cummings et al., 2014; Gauthier et al., 2016; Vandenberghe et al., 2016; Anderson et al., 2017; Carroll, 2017; Coleman and Mastroeni, 2017; Honig et al., 2018). Additionally, amyloid imaging demonstrates that many normal patients can show Aβ plaque deposits, or that AD patients can show low Aβ plaque load (Edison et al., 2007; Li et al., 2008), while in the brain of elderly non-demented patients the distribution of Aβ plaques can be as extensive as that of dementia patients (Davis et al., 1999; Fagan et al., 2009; Price et al., 2009; Chételat et al., 2013). Together, these data suggest that Aβ deposition might be a phenomenon of aging or a consequence of an upstream event and not the causative reason for AD, with scientists now questioning the validity of the amyloid hypothesis (Hardy and Selkoe, 2002; Tanzi and Bertram, 2005; Chételat, 2013; Herrup, 2015).

An important evidence against the amyloid hypothesis is the recent discovery that prior to the accumulation of Aβ depositions, and much earlier than the observation of hippocampal cell death, there is evidence from both an early-AD genetic mouse model (Tg2576) as well as from late-onset patients that the dopamine neurons in the Ventral Tegmental Area (VTA) are compromised (Nobili et al., 2017; De Marco and Venneri, 2018; Serra et al., 2018). The VTA is the origin of tyrosine hydroxylase positive (TH^+^) axons forming the mesocorticolimbic dopaminergic pathway [Figure 1A; (Gasbarri et al., 1994; Björklund and Dunnett, 2007)], projecting primarily to the prefrontal cortex, hippocampus, nucleus accumbens (NAc), olfactory bulb, and amygdala. Degeneration of VTA dopamine neurons in Tg2576 mice is evident as early as 3 months of age and is accompanied by significant levels of local inflammation, while cell death worsens with age (Nobili et al., 2017; D'Amelio and Nisticò, 2018). The degeneration of dopamine neurons results in lower dopamine outflow in the hippocampus and NAc and correlates temporally with impairments in hippocampal neuronal excitability, synaptic plasticity, memory, and reward performance (Nobili et al., 2017; Cordella et al., 2018). Other brain regions containing TH^+^ neurons, in particular the Substantia Nigra pars compacta (SNpc) and Locus Coeruleus (LC), are intact in Tg2576 mice at least at early stages (Nobili et al., 2017), suggesting that the observed degeneration primarily affects the VTA. In line with data from Tg2576 mice, TH^+^ neuron loss has been associated to Aβ load in two other frequently used AD models, the 3 × Tg-AD and APPswe/PS1ΔE9 mice (Liu et al., 2008; Moreno-Castilla et al., 2016), highlighting the role of the VTA and LC in the disease progression. Consistently with the aforementioned observations, pharmacological manipulations aimed at increasing the dopaminergic transmission in the hippocampus and cortex can improve synaptic functions, cognitive impairment and memory deficits in AD patients and in experimental models, emphasizing the critical role of dopamine for proper brain function (Monteverde et al., 1990; Tsunekawa et al., 2008; Himeno et al., 2011; Jürgensen et al., 2011; Koch et al., 2011, 2014; Guzmán-Ramos et al., 2012; Pazini et al., 2013; Martorana and Koch, 2014; Nobili et al., 2017).

**Figure 1:**
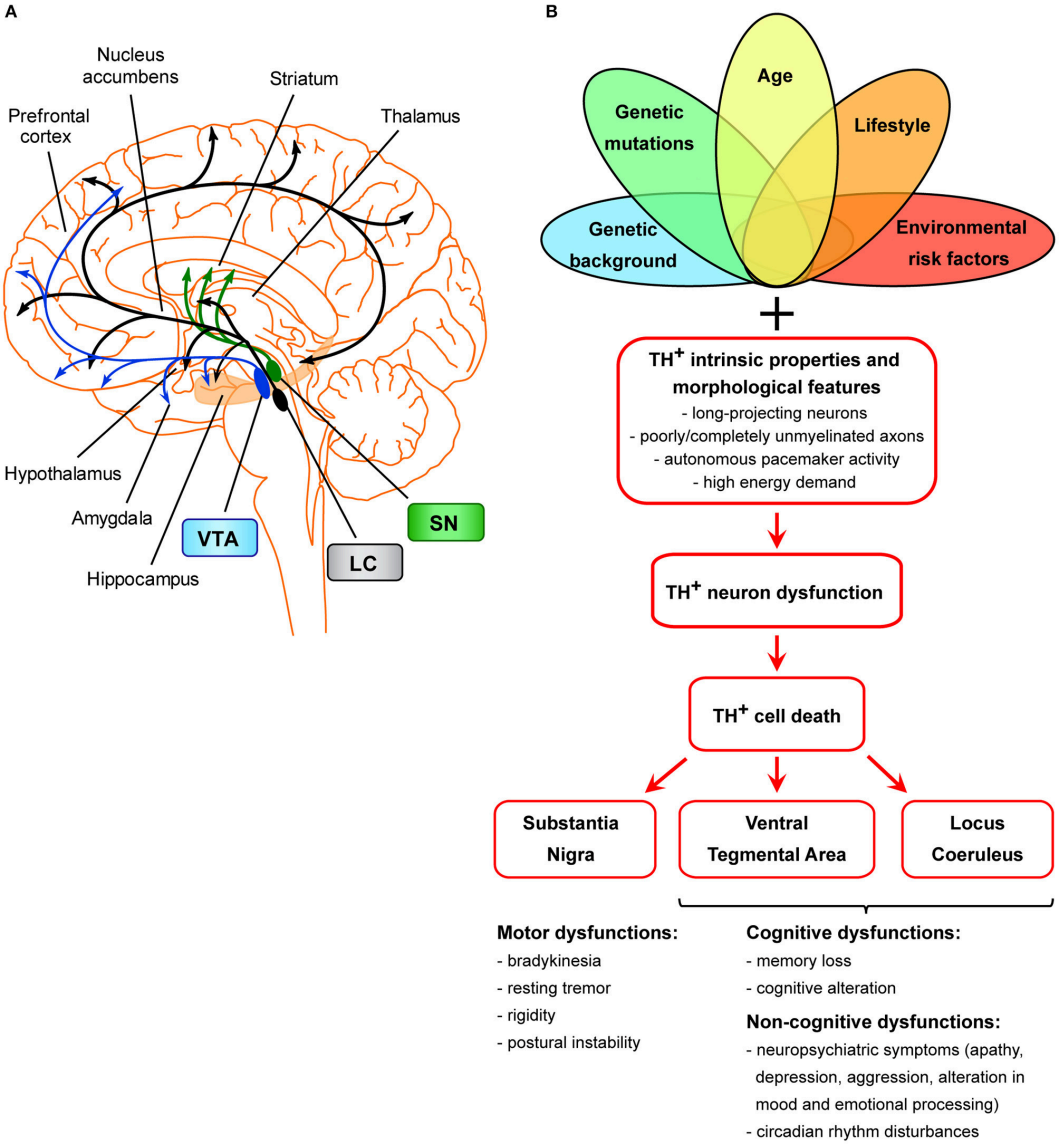
Scheme of the unifying hypothesis of dopamine neuron loss in neurodegenerative diseases. **(A)** Scheme of the dopaminergic projections arising from the VTA, SNpc, and LC. The dopaminergic neurons of the SNpc project mainly to the dorsal striatum (caudate and putamen), mediating the control of voluntary movements via the direct and indirect pathways (Calabresi et al., 2014). Degeneration of these neurons is the main cause of motor symptoms in PD (Surmeier, 2007). LC TH^+^ neurons innervate most of the cortex and subcortical regions and have been shown to be important for cognitive and non-cognitive functions, memory consolidation, attention, sleep-wake cycle, mood, and emotional processing (Palmer et al., 1987; Sara, 2009; Robertson et al., 2013; Kempadoo et al., 2016; Roy et al., 2016; Takeuchi et al., 2016; Borodovitsyna et al., 2017). The VTA is the origin of TH^+^ axons forming the mesocortical and the mesolimbic dopaminergic pathways, together called the mesocorticolimbic system (Gasbarri et al., 1994; Björklund and Dunnett, 2007). The mesocorticolimbic system projects primarily to the prefrontal cortex, hippocampus, NAc, olfactory bulb, amygdala, and anterior cingulate cortex. This system is implicated in motivated behaviors, reward and emotional processing, memory, and learning. Loss of VTA and LC TH^+^ neurons in AD is thought to be the basis for memory deficits, cognitive, and neuropsychiatric symptoms (Kelly et al., 2017; Nobili et al., 2017; D'Amelio et al., 2018a; Serra et al., 2018), while loss of VTA and LC TH^+^ neurons in PD is linked to the appearance of neuropsychiatric symptoms and dementia (Jellinger, 1991; German et al., 1992; Surmeier, 2007; Costa et al., 2012; Alberico et al., 2015; Caminiti et al., 2017). **(B)** Scheme of TH^+^ cell death in age-related neurodegeneration. Several factors (such as genetic background, genetic mutations, age, environmental or lifestyle risk factors), either alone or in combinations, might selectively dispose TH^+^ neurons of a specific area (SNpc, VTA, LC) to degenerate. Moreover, these neurons are more prone than others to cell death due to features that make them particularly sensitive: these cells are long-projecting neurons with poorly or completely unmyelinated axons that provide massive synaptic innervations in projecting areas (Braak et al., 2006), they have autonomous pacemaker activity due to which they have high requirements for energy and therefore also require efficient mitochondrial function. From a clinical point of view, the manifestation of clinical symptoms is strictly associated with the functions of SNpc-, VTA-, and/or LC-projecting areas.

Due to the strong involvement of LC TH^+^ neurons in prodromal AD subjects (Kelly et al., 2017), a clinical study made an effort to consolidate the events of TH^+^ neuron degeneration in AD by clarifying the temporal evolution of changes in the VTA and LC. The study recruited a large sample of patients with either amnestic mild cognitive impairment (MCI) or late-onset AD and healthy non-demented subjects and used resting-state functional MRI to assess the functional connectivity between the VTA or LC and other brain areas (Serra et al., 2018). The rational was that loss of neurons within a specific nucleus such as the VTA or LC would result in reduced connectivity between that nucleus and its projection areas. In line with the data from Tg2576 mice (Nobili et al., 2017), the study showed a significant disconnection between the VTA and the thalamus, medial-temporal structures and the parietal lobe in MCI patients, while these patients show no significant changes in LC connectivity. LC disconnection was only observed in late-AD patients and is distributed to the posterior parietal cortex (Serra et al., 2018). In MCI patients the VTA disconnection is predominant with hippocampal and parietal regions, while in AD patients it involves all nodes of the default-mode network (Gili et al., 2011; Bozzali et al., 2012; Serra et al., 2018). A second study used structural MRI to show that the volume of the VTA can be associated with the typical clinical markers of AD, namely the hippocampal size and memory index (D'Amelio et al., 2018b; De Marco and Venneri, 2018). The main finding was that a smaller VTA corresponds to worse indices of memory and hippocampal size. The authors did not find association between the SNpc and the size of the hippocampus in AD patients, excluding a generic reduction of the midbrain volume and confirming a prominent involvement of the VTA (De Marco and Venneri, 2018). These studies suggest that the VTA volume or connectivity might represent prodromal clinical markers, with the potential of predicting the conversion from healthy state to clinical AD.

From a clinical point of view, the VTA degeneration in AD could be particularly relevant for the manifestation of neuropsychiatric symptoms since mesocorticolimbic dopamine broadly regulates non-motor behavior like motivation, reward, pleasure, attention, emotional processing, and mood conditioning [Figure 1B; (Grace et al., 2007; Bromberg-Martin et al., 2010; Russo and Nestler, 2013)]. In fact, although AD is considered principally a cognitive and memory disorder, almost all patients develop neuropsychiatric symptoms and personality changes that could be explained by the reduced connectivity of the VTA with the default-mode network (Serra et al., 2018). These symptoms, particularly depression, aggression, personality changes and circadian rhythm disturbances, are frequently observed very early in AD (Lyketsos et al., 2011; Masters et al., 2015; Ismail et al., 2016; Alves et al., 2017; Musiek et al., 2018) and persist in parallel with the deterioration of cognitive function. Depression and apathy are most frequent in people with MCI and early AD, although the development of verbal and physical agitation is also quite evident. As the disease progresses, delusions, hallucinations, and aggression become more frequent, while apathy becomes the most persistent neuropsychiatric symptom throughout AD progression. Additionally, population studies show that circadian rhythm disturbances and the frequency of mood symptoms are much higher in people with AD or MCI compared to the general population (Lyketsos et al., 2002, 2011; Taragano et al., 2009). In fact, the MCI stage, today recognized as an intermediate state of clinical dementia (Petersen, 2009), is particularly important for early intervention because MCI patients progress to AD at a much higher rate if they show depression (Taragano et al., 2009). Direct comparison of patients with and without neuropsychiatric symptoms confirmed that patients with apathy, depression or anxiety have more severe functional disconnection between the VTA and the default-mode network compared to patients that lacked such symptoms (Serra et al., 2018). Surprisingly, besides AD, deficits in the mesolimbic dopamine system are considered the leading cause of neuropsychiatric symptoms and cognitive dysfunction occurring also in Parkinson's disease (PD). Indeed the VTA dopamine neuron loss has been associated to several non-motor symptoms which may precede parkinsonism by many years, reflecting the insurgence of dementia, anxiety and/or depression (Elgh et al., 2009; Gustafsson et al., 2015; Castrioto et al., 2016). In line with this, today it is generally recognized that, although neurodegenerative events affect more severely the nigrostriatal dopamine system in PD, the mesolimbic system is also compromised, even if to a lesser degree (Jellinger, 1991; Alberico et al., 2015; Caminiti et al., 2017). In fact, several analysis of PD brains showed about 50% loss of TH^+^ neurons in the A10 region corresponding to the VTA (Hirsch et al., 1988; German et al., 1989; Alberico et al., 2015).

Thus, it appears that the vulnerability of midbrain dopamine neurons is a common feature both in PD and AD, and the appearance of neuropsychiatric symptoms can be an important prognostic tool.

## A Unifying Hypothesis of Dopamine Cell Death in Age-Related Neurodegeneration?

Although the Tg2576 mouse is a genetic model of AD (Hsiao et al., 1996) and early-onset AD is overall attributed to mutations in specific genes (Blennow et al., 2006), the experimental evidence that the first neurons to degenerate in Tg2576 mice are the dopaminergic neurons in the VTA (Nobili et al., 2017) suggests that these neurons are more susceptible than others to cell death. This indicates that several factors other than APP overexpression could contribute to their susceptibility. This observation is in line with the fact that alterations in the functional connectivity or volume of the VTA are observed in late-onset AD patients, overall suggesting that the susceptibility of dopamine neurons is a common theme in both early- and late-onset AD (D'Amelio et al., 2018a).

The increased vulnerability of VTA dopaminergic neurons in AD could be considered a symmetrical picture to the increased vulnerability of SNpc dopaminergic neurons in PD, or the degeneration of TH^+^ neurons in the LC in both AD and PD patients (German et al., 1992; Liu et al., 2008; Kelly et al., 2017; D'Amelio et al., 2018a). In fact, LC neurons might be considered as “atypical” dopaminergic cells that co-express dopamine with noradrenaline (Kempadoo et al., 2016; Takeuchi et al., 2016; Beas et al., 2018). These three TH^+^ regions (SNpc, VTA, LC) project to a widespread number of brain areas, each with a specific function (Figure 1B). Therefore, it is logical to assume that the different manifestation of clinical symptoms in AD, PD, or even PD with dementia, would be dependent on the brain areas that suffer mostly from the dopamine loss and on the degree of TH^+^ neuron degeneration.

What, then, causes TH^+^ cell death in age-related neurodegeneration? These neurons in SNpc, VTA and LC share common features that can increase their susceptibility. They are long-projecting neurons with poorly or completely unmyelinated axons that provide massive synaptic innervations in projecting areas (Braak et al., 2006). As such, efficient mechanisms of axonal transport and autophagy are essential for removal of damaged mitochondria and for regeneration of synaptic components. Additionally, these cells have autonomous pacemaker activity that might contribute to their vulnerability, due to the high requirements for energy and therefore efficient mitochondrial function.

These functional and structural features could lead to further compromise by the metabolism of dopamine, mitochondrial dysfunction, oxidative stress, impaired protein degradation, metal homeostasis, neurochemistry of metalloproteins and neuroinflammation (Schapira, 2001; Liss et al., 2005; Surmeier, 2007; Lim et al., 2016; Burbulla et al., 2017; Mor et al., 2017; Adlard and Bush, 2018). All these factors have been extensively studied in PD to explain the loss of SNpc neurons [reviewed in (Sulzer, 2007; Surmeier et al., 2011; Tambasco et al., 2018)] but their contribution to the vulnerability of VTA or LC dopamine neurons is yet to be defined. Thus, mitochondrial stress, deficits in calcium buffering or increased oxidation by dopamine by-products might all worsen the ability of dopamine neurons to defend themselves from internal or external risk factors.

Unlike the common features mentioned above, these neurons also have specific intrinsic characteristics that differentiate them from each other, such as different potassium and calcium conductances, calcium-binding proteins, or genetic profiling (Neuhoff et al., 2002; Liss et al., 2005; Lammel et al., 2008; Guzman et al., 2009; Khaliq and Bean, 2010; Panman et al., 2014; Poulin et al., 2014). These characteristics, either alone or in combinations also with genetic mutations, age, environmental, or lifestyle risk factors, can induce a selective vulnerability of different SNpc, VTA, and LC TH^+^ neuron subpopulations to cell death. Indeed, the enormous intrinsic variability in dopamine neuron subpopulations (Neuhoff et al., 2002; Lammel et al., 2008; Roeper, 2013; Krashia et al., 2017) might result in different pathophysiological responses to metabolic, genetic, environmental or lifestyle risk factors, and in this way might contribute to the differential vulnerability.

To summarize, in recent years increasing evidence has proven a strong association between deficits in dopaminergic signaling and several cognitive and neuropsychiatric alterations related to AD (Martorana and Koch, 2014; D'Amelio et al., 2018a). Novel knowledge on the vulnerability of VTA dopamine neurons in AD, explaining these deficits, is just starting to emerge. However, the unifying hypothesis of dopamine cell death in age-related neurodegeneration proposed here is highly appealing: lessons learned from PD studies can be essential for disentangling the enigma of dopamine neuron death in AD. We can conclude that, like PD, AD could be considered as a complex disease with multiple etiologies, external and internal, all converging at massive dopamine neuronal loss. This unifying hypothesis of dopamine cell death in age-related neurodegeneration is an interesting hypothesis that merits more thought.

## Author Contributions

PK, AN, and MD equally contribute in writing the article.

### Conflict of Interest Statement

The authors declare that the research was conducted in the absence of any commercial or financial relationships that could be construed as a potential conflict of interest.
